# Platelet‐rich fibrin elicits an anti‐inflammatory response in macrophages in vitro

**DOI:** 10.1002/JPER.19-0216

**Published:** 2019-09-14

**Authors:** Jila Nasirzade, Zahra Kargarpour, Sadegh Hasannia, Franz Josef Strauss, Reinhard Gruber

**Affiliations:** ^1^ Department of Oral Biology Medical University of Vienna Vienna Austria; ^2^ Department of Biochemistry Faculty of Biological Sciences Tarbiat Modares University Tehran Iran; ^3^ Department of Conservative Dentistry School of Dentistry University of Chile Santiago Chile; ^4^ Department of Periodontology School of Dental Medicine University of Bern Bern Switzerland

**Keywords:** anti‐inflammatory agents, arginase, blood platelets, cytokines, macrophages, platelet‐rich fibrin

## Abstract

**Background:**

Platelet‐rich fibrin (PRF) serves as a reservoir of bioactive molecules to support wound healing and bone regeneration. The beneficial action of PRF might involve macrophage polarization from proinflammatory M1 toward pro‐resolving M2 phenotypes. This study aims to evaluate the effect of PRF on macrophage polarization.

**Methods:**

Murine primary macrophages and RAW 264.7 cells were exposed to saliva and lipopolysaccharides (LPS) with and without PRF lysates obtained by repeated freeze‐thawing or the secretome of PRF membranes, termed PRF conditioned medium. The expression of the M1 marker genes interleukin 1β (IL1β) and interleukin 6 (IL6) along with the M2 markers arginase‐1 and chitinase‐like 3 (Chil3 or YM1) were evaluated by real time polymerase chain reaction. Immunoassay and immunofluorescence staining were performed for IL6 and p65 translocation, a subunit nuclear factor kappa‐light‐chain‐enhancer of activated B cells (NF‐kB), respectively.

**Results:**

We report here that PRF lysates and PRF conditioned medium, the latter containing the secretome, greatly decreased the proinflammatory response of primary macrophages and RAW 264.7 cells as indicated by the expression of IL1β and IL6. The anti‐inflammatory activity of PRF lysates was further confirmed by IL6 immunoassay. Moreover, PRF lysates suppressed the translocation of p65 from the cytoplasm into the nucleus after incubation with saliva. In support of M2 polarization, PRF lysates and PRF conditioned medium enhanced the expression of arginase‐1 and YM1 in primary macrophages.

**Conclusion:**

Our results indicate that PRF holds an anti‐inflammatory activity and shifts the macrophage polarization from an M1 toward an M2 phenotype.

## 1 INTRODUCTION

Platelet‐rich fibrin (PRF), a natural autologous scaffold, was introduced almost 20 years ago.^1^ In a clinical setting, PRF is prepared by the centrifugation of blood, with the coagulated plasma being harvested and transferred to a defect site.^1^ PRF meets the basic requirements of a wound dressing through its three‐dimensional fibrin scaffold which is enriched in growth factors and cells such as platelets and leukocytes.^2^, ^3^ There is a growing body of evidence suggesting that PRF improves the clinical outcomes, particularly in implant dentistry.^4^ PRF membranes can increase the width of keratinized mucosa around implants^5^ and preserve the horizontal and vertical ridge dimension at 3 months after tooth extraction.^6^ Although the clinical outcomes of PRF are promising, the underlying cellular mechanism by which PRF contributes to wound healing remains unclear.

Macrophages are critically involved in wound healing^7^ and bone regeneration,^8^ a process that might involve a polarization switch from an M1 proinflammatory to an M2 pro‐resolving phenotype.^9^, ^10^ Macrophages M1 type appear during tissue damage causing inflammation, subsequently M2 types dominate the scene to help tissue repair and remodeling with the ensuing resolution of inflammation.^11^ Wound healing is an active process encompassing lipid mediators with dual anti‐inflammatory and pro‐resolution activities such as lipoxins.^12^, ^13^ These pro‐resolving mediators in conjunction with proinflammatory mediators are required for the recruitment of non‐phlogistic macrophages.^12^, ^13^ Considering that this fibrin scaffold is later populated by immigrating macrophages,^14^ it is plausible to assume that platelets are essential to drive the macrophage polarization.

Even though various preparation containing platelets can suppress an in vitro inflammatory response, this has not been proven for PRF. For example, platelet‐rich plasma lowered the inflammatory response of mouse bone marrow‐derived macrophages and increased the M2 macrophage marker arginase‐1 expression.^15^, ^16^ Platelet‐rich plasma decreased the inflammatory response of human monocyte‐derived dendritic cells and macrophages stimulated with *Aspergillus fumigatus*.^17^ Moreover, leukocyte‐depleted platelet‐rich plasma increased the mannose receptor CD206 in human macrophages suggesting a shift toward M2 phenotype.^18^ These pioneer findings suggest that various preparations containing platelets can control macrophage polarization, particularly the shift from M1 proinflammatory to M2 pro‐resolving phenotypes. However, studies addressing PRF and macrophage polarization have not yet been performed.

In vitro research on macrophage polarization involves murine marrow‐derived macrophages and cell lines such as RAW 264.7, which are exposed to lipopolysaccharides (LPS) and IL4 to push the M1 and M2 phenotype, respectively.^19^, ^20^ Apart from LPS, M1 polarization is caused by saliva.^21^ Among the main markers of M1 macrophages are the inflammatory cytokines IL1 and IL6. M2 macrophages express marker genes such as arginase‐1^22^ and YM1, the latter also known as chitinase‐like 3.^23^ Reports on growth factors and immunologic factors of PRF have revealed the presence of IL6, IL8, IL4, and TGF‐β, all of which can modulate macrophage polarization.^24^ For example, IL6 and IL8 provide an inflammatory environment that favors the formation of M1 macrophages,^25^ whereas particularly IL4^26^ but also TGF‐β skews macrophage polarization toward an M2‐like phenotype.^27^ However, it is not possible to draw conclusions from single factors released upon platelet activation on macrophage polarization by PRF, as the secretome of PRF with its plasma components and other cell types, is complex.

We propose here that PRF modulates macrophage polarization. Based on in vitro models on macrophage polarization, we found that PRF lysates and conditioned medium causes a shift from M1 toward the M2 phenotype.

## 2 MATERIALS AND METHODS

### 2.1 Isolation and culture of murine bone marrow‐derived macrophages and RAW 264.7 cells

BALB/c mice of 6‐ to 8‐weeks old were purchased from Animal Research Laboratories, Himberg, Austria. Bone marrow cells were collected from the femora and tibiae as previously described.^28^ Briefly, mice were sacrificed, and the femora and tibiae of the mice were removed. Bone marrow cells were seeded at 4 × 10^6^ cells/cm^2^ into 12‐well plates and grown for 5 days in Minimum Essential Medium Eagle‐Alpha Modification^1^ (α MEM) supplemented with 10% fetal bovine serum^2^, antibiotics^1^ and with 20 ng/mL macrophage colony‐stimulating factor (M‐CSF).^3^ RAW 264.7 macrophage‐like cells^4^ were expanded in growth medium and seeded at 1 × 10^5^ cells/cm^2^ into 12‐well plates. Bone marrow macrophages and RAW 264.7 were exposed to the respective treatments for another 24 hours under standard conditions at 37°C, 5% CO_2_, and 95% humidity.

### 2.2 Preparation of PRF lysates and conditioned medium

PRF membranes were prepared after the approval of the ethics committee of the Medical University of Vienna (1644/2018) and volunteers signed informed consent forms. All experiments were performed in accordance with relevant guidelines and regulations and were conducted in accordance with the Helsinki Declaration of 1975, as revised in 2000. Venous blood was collected at the University Clinic of Dentistry from six healthy volunteers, each donating six plastic glass‐coated tubes^5^ allowing spontaneous blood coagulation. Platelet‐rich fibrin (PRF) membranes were produced using a protocol of 1,570 RPM for 12 minutes (RCF‐max = 400 g). PRF membranes were produced using a centrifuge device with universal swing‐out rotors^6^ (146 mm at the max) using 10‐mL glass‐coated plastic tubes. The PRF clot was separated from the remaining red thrombus and compressed between two layers of dry gauze. Each PRF membrane was transferred into serum‐free medium (1 cm PRF/mL) and exposed to two cycles of freeze‐thawing and sonication^7^ as reported for human platelet lysate.^29^^‒^ 31 After centrifugation^8^ at 15,000 × g for 10 minutes, the supernatants of at least three PRF membranes were harvested, pooled, and stored at −20°C before the in vitro analysis. In indicated experiments, PRF membranes were transferred into serum‐free medium (1 cm PRF/mL) and placed into an incubator at 37°C to allow a natural release of growth factors into the culture media, similarly as previously described.^32^ At 24 hours the conditioned medium was collected.

### 2.3 Stimulation of murine bone marrow‐derived macrophage and RAW 264.7 cells with soluble extract of PRF

Bone marrow‐derived macrophages and RAW 264.7 cells were exposed to 5% saliva and LPS from *Escherichia coli* 0111: B4^1^ at 100 ng/mL to reach M1 phenotype, and recombinant mouse IL4^9^ at 10 ng/mL to obtain M2 phenotype. In the case of PRF anti‐inflammatory effect, PRF lysates at 30% (v/v) were added to cells in the presence of LPS or saliva and gene expression changes of IL1β and IL6 were evaluated. To analyze the potential of PRF lysate to cause an M2 phenotype, arginase‐1 (ARG1) and YM1 gene expression was measured in primary macrophages treated with PRF, and in indicated experiments also in combination with saliva and LPS. After overnight incubation, expression changes of M1/M2 marker genes were determined. To examine the influence of TGF‐β signaling, the inhibitor of TGF‐β receptor type I kinase, SB431542^10^ was used at 10 µM. Cells were also treated with different concentrations of PRF lysates up to 50% before gene expression was analyzed. In indicated experiments, cells were exposed to PRF conditioned medium instead of PRF lysates.

### 2.4 RT‐PCR analysis and immunoassay

After overnight stimulation total RNA was isolated^11^ and reverse transcription was performed.^10^ Polymerase chain reaction was performed^12^ with a Real‐Time PCR Detection System.^13^ Primer sequences were mIL1β‐F: AAGGGTGCTTCCAAACCTTTGAC, mIL1β‐R: ATACTGCCTGCCTGAAGCTCTTGT; mIL6‐F: GCTACCAAACTGGATATAATCAGGA, mIL6‐R: CCAGGTAGCTATGGTACTCCAGAA; mARG1‐F: GAATCTGCATGGGCAACC, mARG1‐R: GAATCCTGGTACATCTGGGAAC; mYM1‐F: CATTCCAAGGCTGCTACTCA, mYM1‐R: TCATGACCTGAATATAGTCGAGAGA; mGAPDH‐F: AACTTTGGCATTGTGGAAGG, mGAPDH‐R: GGATGCAGGGATGATGTTCT mALOX5‐F: AGCATGAAAGCAAGGCGCATA, mALOX5‐R: GTACGCATCTACGCAGTTCTG; mALOX12‐F: TCCCTCAACCTAGTGCGTTTG; mALOX12‐R: GTTGCAGCTCCAGTTTCGC; mALOX15‐F: GGCTCCAACAACGAGGTCTAC; mALOX15‐R: AGGTATTCTGACACATCCACCTT; mIL10‐F: GCCTTATCGGAAATGATCCA; mIL10‐F: TCTCACCCAGGGAATTCCAAA. The mRNA levels were calculated by normalizing to the housekeeping gene GAPDH using the ΔΔCt method. Supernatants were analyzed for IL‐6 secretion by immunoassay according to the manufacturer's instruction.^14^

### 2.5 Immunostaining

Immunofluorescent analysis of NF‐kB p65 was performed on RAW 264.7 plated onto glass slides^15^ treated with 5% saliva for 30 minutes before being exposed to 50% soluble extract of PRF. Cells were fixed in paraformaldehyde and blocked in 1% BSA and 0.3% Triton in PBS at room temperature for 1 hour. Cells were subsequently incubated with NF‐kB p65 primary antibody^16^ (1:100), overnight. Alexa 488 secondary antibody^17^ (1:200) was applied for 1 hour. Cells were washed and nuclei stained with DAPI^18^ (100 ng/mL) and mounted onto glass slides. Fluorescent images were captured at 100× in oil immersion using a fluorescent microscope.^19^

### 2.6 Statistical analysis

All experiments were performed three to six times. Bars show the mean and standard deviation of the cumulative data from all experiments. Statistical analysis was based on Mann‐Whitney *U* test and Kruskal‐Wallis test with Dunn multiple comparisons correction. Analysis was performed using statistical software.^20^ Significance was set at *P* <0.05.

## 3 RESULTS

### 3.1 PRF lysate exerts a dose‐dependent anti‐inflammatory effect on RAW 264.7 cells

To examine the potential role of PRF on macrophage polarization, RAW 264.7 cells were exposed either to saliva or LPS in the presence or absence of various concentrations of PRF lysates. Dose‐response curves revealed that 30% PRF causes a robust suppression of the proinflammatory marker gene IL6 induced by 5% saliva (see supplementary Figure 1A in online *Journal of Periodontology*) or 100 ng/mL LPS (see supplementary Figure 1B in online *Journal*). Based on the dose‐response curve, further experiments were performed with 30% PRF lysates.

### 3.2 PRF lysate decreases the inflammatory response in bone marrow macrophages and RAW 264.7 cells

**Figure 1:**
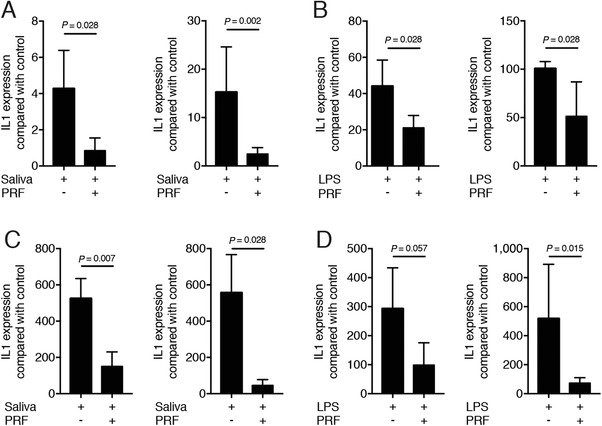
PRF lysate decreases the inflammatory response in bone marrow macrophages and RAW 264.7 cells. Bone marrow macrophages (**A** and **B**) and RAW 264.7 cells (**C** and **D**) were exposed to 30% PRF lysates in the presence of (**A** and **C**) 5% saliva and (**B** and **D**) 100 ng/mL LPS. Data show the x‐fold changes of IL1β and IL6 gene expression; n = 4 to 6. Statistical analysis was based on a Mann‐Whitney *U* test, and *P* values are indicated

**Figure 2:**
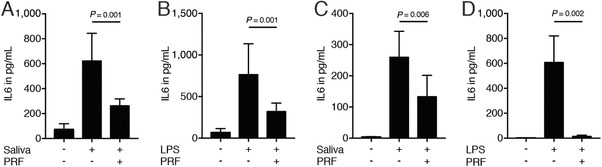
PRF lysate decreases IL6 translation in bone marrow macrophages and RAW 264.7 cells. Bone marrow macrophages (**A** and **B**) and RAW 264.7 cells (**C** and **D**) were exposed to 30% PRF lysates in the presence of 5% saliva (**A** and **C**) and 100 ng/mL LPS (**B** and **D**). Data show the IL6 levels in the supernatant; n = 4 to 6. Statistical analysis was based on a Mann‐Whitney *U* test, and *P* values are indicated

Next, primary macrophages from murine bone marrow cells and RAW 264.7 cells were exposed either to saliva or LPS in the presence or absence of 30% PRF lysates. PRF strongly suppressed the saliva‐ and LPS‐induced inflammation in primary macrophages (Figs. 1A and 1B) and RAW 264.7 cells (Figs. 1C and 1D), indicated by the reduced expression of the genes IL1β and IL6. In line with the gene expression data, PRF lysates substantially reduced the IL6 levels provoked by saliva or LPS in primary macrophages (Figs. 2A and 2B) and RAW 264.7 cells (Figs. 2C and 2D), respectively. Altogether, these results suggest that PRF has an anti‐inflammatory effect attenuating an M1 polarization.

### 3.3 PRF lysate attenuates the translocation of NF‐κB from the cytoplasm into the nucleus

**Figure 3:**
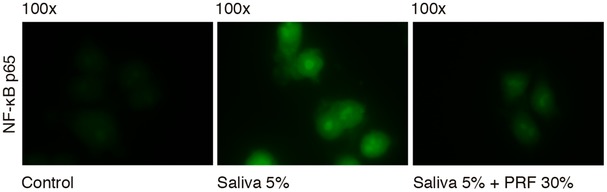
PRF lysate attenuates the translocation of NF‐κB from the cytoplasm into the nucleus. PRF lysate reduces the intracellular translocation of Nf‐kB p65 into nucleus, induced by 5% saliva in RAW 264.7. Immunofluorescence analysis indicates reduced Nf‐kB p65 presence in the nucleus upon the addition of PRF on cells treated with saliva

To further confirm the inhibitory effect of PRF lysates on inflammation, we performed an immunofluorescent analysis of NF‐κB translocation from the cytoplasm into the nucleus. The presence of PRF lysates strongly reduced the NF‐κB p65 signaling activation induced by saliva in RAW 264.7 (Fig. 3). This finding suggests that PRF can modulate the inflammation pathway also at the level of NF‐κB p65 signaling.

### 3.4 PRF lysate induces the formation of alternatively activated macrophages

**Figure 4:**
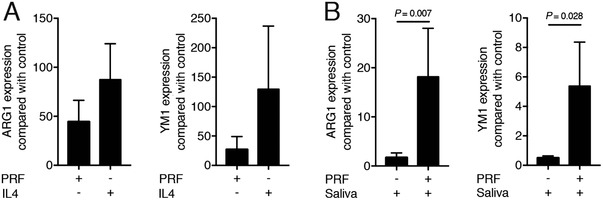
PRF lysate induces the formation of alternatively activated macrophages. Bone marrow macrophages (**A**) were exposed to 30% PRF lysates or 10 ng/mL IL4. Bone marrow macrophage (**B**) cells were exposed to 30% PRF lysates in the presence of 5% saliva. Data show the x‐fold changes of ARG1 and YM1 expression in bone marrow cultures; n = 4 to 6. Statistical analysis was based on a Mann‐Whitney *U* test, and *P* values are indicated

To examine the effect of PRF on alternatively activated M2 macrophages, bone marrow macrophages were cultured either with PRF lysates or with IL4. As with IL4, PRF lysates induced an M2 macrophage polarization indicated by robust expression of ARG1 and YM1 (Fig. 4A). To examine whether PRF pushes an M2 response also under inflammatory conditions, macrophages were cultured with saliva in the absence or presence of PRF lysates. PRF induced an M2 response despite the presence of saliva, indicated by the strong regulation of ARG1 and YM1 (Fig. 4B). Overall, these results suggest that PRF lysate induces the formation of alternatively activated macrophages even under inflammatory conditions.

### 3.5 Growth factors naturally released by PRF membranes suppress inflammation in vitro

**Figure 5:**
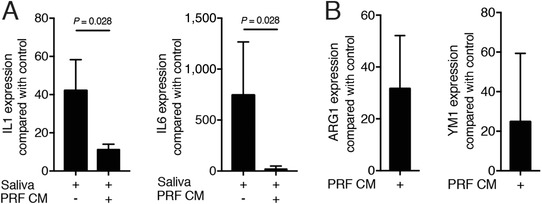
Growth factors naturally released by PRF membranes suppress inflammation in vitro. RAW 264.7 cells (**A**) were exposed to 5% saliva without or with 30% of PRF conditioned medium (CM) which was harvested after 24 hours of incubation. Data show the x‐fold changes of IL1β and IL6 expression in RAW 264.7 cells in the absence or presence of PRF conditioned medium. **B**) ARG1 and YM1 expression in bone marrow macrophages induced by PRF conditioned medium; n = 3 to 4. Statistical analysis was based on a Mann‐Whitney *U* test, and *P* values are indicated

To simulate the natural release of growth factors from PRF membranes, membranes were transferred into culture medium. After 24 hours the conditioned medium (CM) was collected.^32^ In support of the data obtained with PRF lysates, PRF conditioned medium reduced the inflammatory response of RAW 264.7 cells induced by saliva (Fig. 5A). Moreover, PRF conditioned medium caused a substantial increase of ARG1 and YM1 in primary macrophages (Fig. 5B). These observations suggest that factors naturally released from PRF membranes are capable of changing macrophage polarization in vitro.

### 3.6 Blocking of the TGF‐β receptor type I kinase can partially reverse the effects of PRF lysates

**Figure 6:**
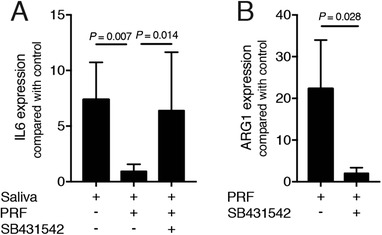
Blocking of the TGF‐β receptor type I kinase with SB431542 reverses the effects of PRF lysates. SB431542 reversed the anti‐inflammatory activity of PRF on bone marrow macrophages exposed to saliva based on the expression of IL6 (**A**). SB431542 hindered the PRF lysate‐induced increase of ARG1 in bone marrow macrophages (**B**); n = 4 to 6. Statistical analysis was based on a Kruskal‐Wallis test with Dunn multiple comparisons correction and Mann‐Whitney *U* test. *P* values are indicated

To understand the possible involvement of TGF‐β signaling in mediating the effects of PRF lysates on macrophage polarization, we blocked TGF‐β receptor type I kinase with SB431542. SB431542 reversed the anti‐inflammatory activity of bone marrow macrophages being exposed to saliva based on the expression of IL6 (Fig. 6A). Moreover, SB431542 hindered the PRF lysate‐induced increase of ARG1 in bone marrow macrophages (Fig. 6B). Thus, part of the activity of PRF lysates is mediated via activation of TGF‐β signaling.

### 3.7 PRF lysates moderately change the expression of lipoxygenases

To examine as to whether murine macrophages release inflammation‐resolving mediators such as lipoxin A4 and resolvin E1 upon PRF stimulation, the expression of the respective lipoxygenases was determined. Supplementary Figure 2 in online *Journal of Periodontology* shows a moderate but significant increase of ALOX5, ALOX12, and ALOX15 when bone marrow macrophages were exposed to PRF lysates. Next, the expression of IL10 was assessed. However, IL10 was downregulated by PRF lysates by roughly 30%, which is surprising considering that IL10 is an anti‐inflammatory cytokine (data not shown). These data suggest that PRF can also modulate the expression of lipoxygenases.

## 4 DISCUSSION

Since the introduction of PRF as an autologous temporary extracellular matrix of coagulated PRP,^1^ numerous attempts were made to support the use of PRF for clinical application. Despite the low number of randomized controlled clinical trials,^33^ individual studies underline the clinical potential of local application of PRF in regenerative dentistry, and particularly in implant dentistry.^33^ For example, a well‐designed clinical research provided the first insights into the potential of PRF in preserving the alveolar ridge after tooth extraction.^6^ The underlying molecular and cellular mechanisms, however, remain unknown. Considering that induction of M2 macrophages can prevent alveolar bone loss in periodontitis models,^34^ and PRF is used for periodontal tissue engineering,^35^ the question then arises whether PRF can tune macrophage polarization. In support of this assumption, we report here that PRF can modulate macrophage polarization in vitro by shifting the M1 toward an M2‐like phenotype.

Our results match with those observed in earlier studies on other platelet preparations such as platelet‐rich plasma. Platelet‐rich plasma holds an anti‐inflammatory effect on murine RAW 264.7 cells and primary macrophages and also induces the expression of ARG1.^15^, ^16^ Furthermore, PRP decreased the inflammatory response of human dendritic cells and macrophages stimulated with *A. fumigatus* 17 and leucocyte‐depleted PRP increased the M2 marker mannose receptor CD206 in human macrophages.^18^ We thus extend previous observations obtained with platelet‐rich plasma but focusing mainly on the clinical approach using PRF membranes.^16^ We show here that PRF lysates produced by freeze‐thawing the membranes^31^ and PRF conditioned medium both decreased the inflammatory response of macrophages. Moreover, they increased ARG1 and YM1 expression, suggesting a shift from M1 toward M2 polarization. Hence, the novelty we provide here is the use of PRF membranes that are routinely used in a clinical setting.

We then focused on the characterization of the PRF‐released factors that may account for our observations. It is possible that the anti‐inflammatory activity and the enhanced expression of M2 marker genes are controlled either by the same or different factors. Considering the high amount of TGFβ in the PRF,^36^ and knowing the capacity of TGFβ1 to modulate M1 and M2 polarization,^27^ we used the TGF‐β receptor type I kinase inhibitor SB431542 to block the respective signaling pathway. Notably, SB431542 substantially modulated the anti‐inflammatory activity and reversed the ARG1 expression in bone marrow macrophages exposed to PRF lysates. Another factor that may assist the M1‐to‐M2 transition and the subsequent anti‐inflammatory activity is the generation of pro‐resolving lipid mediators as PRF increased the expression of all three lipoxygenases.^37^ Altogether, our data indicate that the transition between M1 and M2 occurs partially due to an activation of TGF‐β in PRF^38^ and is likely supported through the production of pro‐resolving lipid mediators. It is unlikely that IL4 from platelets induces the ARG1 expression as PRF contains ≈10 to 50 pg/mL,^39^ which is three decimal less concentrated than the 10 ng/mL that we use to induce the expression of M2 marker genes. Certainly, there is a need to further elucidate the PRF‐derived factors that are responsible for the polarization shift of M1 toward the M2 phenotype.

The clinical relevance of the findings supports PRF as a simple chair‐side therapeutic approach to support a macrophage‐based friendly healing environment. For instance, enhancing M2 macrophages improves bone regeneration in a fracture model.^40^ Moreover, M2 macrophages are highly abundant during the ossification phase in fracture healing.^41^ Macrophages tightly control the biomaterial‐host events during the osseointegration of dental implants.^42^, ^43^ A recent systematic reviews by our group revealed that the use of PRF during implant placement increased implant stability during the early phases of osseointegration.^33^ As dental implants activate the immune system during the early stages of osseointegration,^44^ it is plausible that an M2 polarization by PRF may reduce the time lag for initiation of bone formation. There is clearly a demand on research if the clinical effects of PRF can account for the polarization of local macrophages toward the M2 phenotype.

Another interesting clinical aspect is the macrophage polarization in the context of peri‐implantitis. In tissue samples retrieved from patients with peri‐implantitis M1 macrophages are predominant.^45^ A controlled M1‐to‐M2 transition by the use of pro‐resolving agents might restrict the inflammatory response and consequently suppress the detrimental events induced by peri‐implantitis, as shown in mouse calvarial models.^46^ This mechanism might also explain the positive outcomes using PRF in conjunction with open flap debridement in patients with peri‐implantitis.^33^, ^47^ The clinical evidence is still lacking and therefore the clinical translation of the present results should be interpreted with caution. Much of the knowledge is based on in vitro and preclinical studies; nonetheless, they provide the basic principles for novel strategies to be applied in clinical practice.

There are more open questions that span the gap between basic research and the clinical application. Since g‐force and centrifugation time affect the composition of PRF,^48^ a systematic approach^49^ on how the various forms of PRF affect the in vitro macrophage polarization is warranted. Further research should unravel the involvement of macrophages, particularly the M1 to M2 shift by PRF membranes in a clinical scenario. It is worth to mention that we have used an in vitro approach with murine macrophages. Consequently, it should be examined whether human‐derived macrophages respond similarly to PRF lysates. We also have to consider that regeneration of extraction sockets and osseointegration follow evolutionally conserved principles that can only be slightly pushed but not changed fundamentally. The ideal control for the PRF research should be a natural blood clot that, surprisingly, cannot easily be simulated in vitro. Knowledge gained by comparing in vitro effects of a natural blood clot versus PRF membranes will help to address this issue.

## 5 CONCLUSION

Our in vitro observations indicate that PRF holds an anti‐inflammatory activity and shifts the macrophage polarization from M1 toward M2 phenotype.

## AUTHOR CONTRIBUTIONS

Reinhard Gruber and Franz Josef Strauss contributed to conception and design; contributed to acquisition, analysis, and interpretation; drafted manuscript; critically revised manuscript; gave final approval; agreed to be accountable for all aspects of work. Jila Nasirzade and Zahra Kargarpour contributed to conception and design, contributed to acquisition, analysis, and interpretation; critically revised manuscript; gave final approval; agreed to be accountable for all aspects of work. Sadegh Hasannia contributed to acquisition, analysis, and interpretation; critically revised manuscript; gave final approval; agreed to be accountable for all aspects of work.

## Supporting information

Supporting Information

Supporting Information

## Footnotes

1 Sigma Aldrich, St. Louis, MO.

2 Bio&SELL, Nuremberg, Germany.

3 Prospec, Ness‐Ziona, Israel.

4 LGC Standards, Wesel, Germany.

5 BD Vacutainer Ref 367896, BD, Plymouth, UK.

6 Z 306 Hermle Universal Centrifuge, Wehingen, Germany.

7 Sonopuls 2000.2, Bandelin electronic, Berlin, Germany.

8 Eppendorf, Hamburg, Germany.

9 ProSpec, Ness Ziona, Israel.

10 Calbiochem, Merck, Billerica, MA.

11 Blirt S.A., Gdańsk, Poland.

12 Bioline, London, UK.

13 Bio‐Rad Laboratories, Hercules, CA.

14 R&D Systems, Minneapolis, MN.

15 Merck, Darmstadt, Germany

16 Cell Signaling Technology, Denver, MA.

17 Santa Cruz Biotechnology, Dallas, TX.

18 Roche Diagnostics, Mannheim, Germany.

19 Euromex, Oxion fluorescence, Arnhem, Netherlands

20 GraphPad Software, La Jolla, CA.
