# Supplementary material for: Platelet‐rich fibrin elicits an anti‐inflammatory response in macrophages in vitro
Source: J Periodontol. 2019 Sep 14;91(2):244–52. doi: 10.1002/JPER.19-0216 (PMC7065136; doi:10.1002/JPER.19-0216)
Supplement: Supplementary file 1 — Supporting Information [file JPER-91-244-s001.pdf]

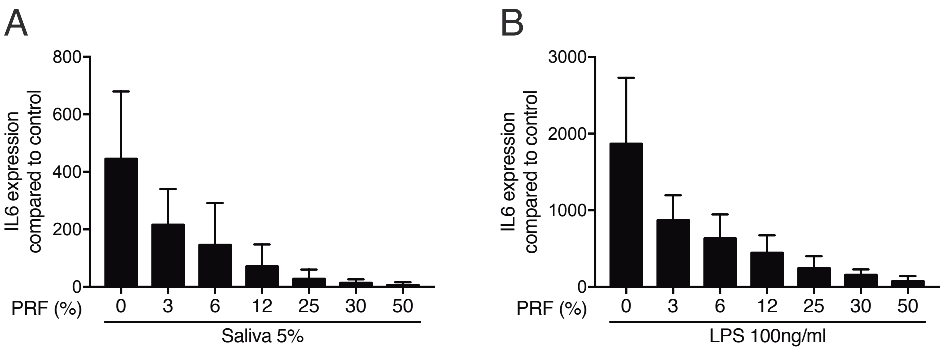

Supplementary Figure 1. PRF lysate exerts a dose-dependent anti-inflammatory effect on RAW 264.7 cells. RAW 264.7 cells were exposed to the indicated concentrations of PRF lysates up to 50% in addition to treatment with (A) 5% saliva and (B) LPS at 100ng/ml. Data show the x-fold changes of IL6 expression in RAW 264.7 cells treated with saliva and LPS. Experiments show the means and standard deviation of three independent experiments.
