# Supplementary material for: Platelet‐rich fibrin elicits an anti‐inflammatory response in macrophages in vitro
Source: J Periodontol. 2019 Sep 14;91(2):244–52. doi: 10.1002/JPER.19-0216 (PMC7065136; doi:10.1002/JPER.19-0216)
Supplement: Supplementary file 2 — Supporting Information [file JPER-91-244-s002.pdf]

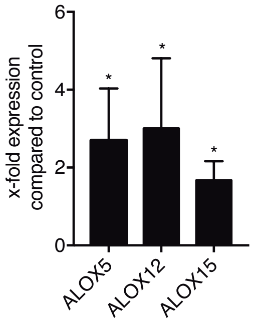

Supplementary Figure 2. PRF lysates increases the expression of lipoxygenases. Bone marrow macrophages were exposed to 30% PRF lysates Data show the x-fold changes of ALOX5, ALOX 12 and ALOX15 compared to unstimulated controls. N=4. Data represent the mean ± SD, *P < 0.05, by two-tailed Mann-Whitney test.
